# Chondrogenic Potential of Human Dental Pulp Stem Cells Cultured as Microtissues

**DOI:** 10.1155/2021/7843798

**Published:** 2021-09-07

**Authors:** Rubén Salvador-Clavell, José Javier Martín de Llano, Lara Milián, María Oliver, Manuel Mata, Carmen Carda, María Sancho-Tello

**Affiliations:** ^1^Department of Pathology, Faculty of Medicine and Dentistry, University of Valencia, Valencia, Spain; ^2^INCLIVA Biomedical Research Institute, Valencia, Spain; ^3^Biomedical Research Networking Center on Bioengineering, Biomaterials and Nanomedicine (CIBER-BBN), Spain

## Abstract

Several tissue engineering stem cell-based procedures improve hyaline cartilage repair. In this work, the chondrogenic potential of dental pulp stem cell (DPSC) organoids or microtissues was studied. After several weeks of culture in proliferation or chondrogenic differentiation media, synthesis of aggrecan and type II and I collagen was immunodetected, and SOX9, ACAN, COL2A1, and COL1A1 gene expression was analysed by real-time RT-PCR. Whereas microtissues cultured in proliferation medium showed the synthesis of aggrecan and type II and I collagen at the 6^th^ week of culture, samples cultured in chondrogenic differentiation medium showed an earlier and important increase in the synthesis of these macromolecules after 4 weeks. Gene expression analysis showed a significant increase of COL2A1 after 3 days of culture in chondrogenic differentiation medium, while COL1A1 was highly expressed after 14 days. Cell-cell proximity promotes the chondrogenic differentiation of DPSCs and important synthesis of hyaline chondral macromolecules.

## 1. Introduction

Nowadays, the incidence of articular pathologies is dramatically rising. Factors such as advanced age of the population, illnesses, lifestyle, or trauma can lead to damage on the articular cartilaginous tissue [1]. Hyaline cartilage extracellular matrix (ECM) is a highly hydrated and gelatinous one, due to the glycosaminoglycan (GAG) component, mainly aggrecan, and the glycoproteins content [2, 3]. In this tissue, type II collagen is the most characteristic fibrous component of the ECM [4]. In addition, articular cartilage has a low regenerative capability due to its physiological characteristics, the absence of vascularization, perichondrium or MSC, and the limited proliferative capability of mature chondrocytes [5]. Thus, the response of joint cartilage to damage is usually the formation of a fibrous scar composed of type I collagen and fibroblast-type cells [6]. This low capability of autonomous regeneration has put cartilage in the spotlight of biomedical research, being currently one of the goals to be achieved [3, 7, 8]. In larger lesions, the mosaicplasty technique can be performed [9], but joint arthroscopic surgery is required for this procedure, since it is used in lesions larger than 2 cm^2^. Furthermore, cartilage cylindrical grafts have to be harvested from a healthy cartilage from a nonweight bearing area. The results obtained are favourable in some cases, with optimal chondral regeneration [9]. However, although classic therapies or surgical treatments (such as mosaicplasty) have been developed with acceptable results in the short term, they have frequently shown fibrocartilage formation in the repaired damage in the long term, and for this reason, they are applied only in a limited number of patients [10, 11].

Tissue engineering (TE) focuses on developing substitutes that help to repair tissue injuries, attempting to achieve optimal physiological and mechanical properties similar to the native tissue [1]. For articular cartilage lesions, this new interdisciplinary science is based on the use of cells, biomaterials, and stimulants such as growth factors [12–16]. Examples of the most popular techniques in which the use of cells is fundamental are Autologous Chondrocyte Implantation (ACI) and Matrix-induced Autologous Chondrocyte Implantation (MACI) techniques [17–20]. However, it has been shown that the use of chondrocytes entails problems associated with the isolation and proliferation procedures and the cellular dedifferentiation [21, 22].

The use of mesenchymal stem cells (MSCs), instead of chondrocytes, is an alternative for TE procedures [21, 23–25]. The plasticity and differentiation potential of these cells has been demonstrated using differentiation techniques [5, 26–31] and also their high proliferation capability when compared to chondrocytes [32–34]. Dental pulp stem cells (DPSCs) have become one of the most common stem cells used in several studies [25, 35^]^, because of their easy accessibility, plasticity, high proliferative ability, and their multiple differentiation capability (chondrogenic, odontogenic, and neurogenic, among others) compared with other MSCs from bone marrow or adipose tissue origin [36–38]. The use of DPSCs in cartilage tissue repair has been reported by several authors (for a review, see [39]). Furthermore, DPSCs are good candidates for developing allogenic histocompatible cell biobanks for research and future clinical applications due to their aforementioned characteristics and immunomodulatory properties [40, 41]. In addition, the use of growth factors (such as IGF, FGF, or TFG*β* [42–45^]^) as well as physical and environmental conditions [46] can help to improve cellular differentiation. In fact, TGF-*β*1 has an important role in chondrogenic differentiation [29, 47]. Most researchers have chosen chondrogenic *in vitro* predifferentiation of MSCs, as DPSCs, with positive results [47–49]. Despite the extended use of biomaterials [29, 50–53], some researchers have demonstrated that pelleted cell cultures enhance chondrogenic differentiation of MSCs better than hydrogel cultures [54–56]. On the one hand, hydrogels are used to repair damaged cartilage [57–62] and can provide cell-matrix interactions and better mechanical support [52, 63]. However, the hydrogel matrix that encloses cells can affect the formation of cell-to-cell contacts, which in turn can influence cell differentiation [64, 65]. On the other hand, scaffold-free cellular organoids also show similar morphology to native cartilage [63]. The main inconvenience of pelleted cell cultures, or microtissues, is the need of a high cellular density per pellet but is tackled by using MSCs due to their high proliferation capacity [32–34]. For this reason, scaffold-free cell-based therapy could be a new treatment for cartilage repair and regeneration [64–66].

We hypothesize that a 3D arrangement of human DPSCs favours their chondrogenic differentiation. Thus, the aim of this work was to study the capability of human DPSCs cultured in microtissues or cellular organoids to develop a chondrogenic phenotype. To assess cellular differentiation, cell morphology and the expression of characteristic chondral macromolecules and genes were analysed in those cultures.

## 2. Material and Methods

### 2.1. Cell Culture

Human dental pulp stem cells (hDPSCs; Lonza, Switzerland) on passage 4 were seeded on culture flasks with proliferation medium containing alpha minimum essential media (*α*MEM; Gibco, USA) supplemented with 10% foetal bovine serum (Gibco), 1% L-glutamine (Gibco), and 1% penicillin/streptomycin (Gibco) and cultured in a humidified atmosphere incubator at 37°C and 5% CO_2_. Culture media were changed every 2-3 days. Cells were detached from the flask with a 0.25% (*w*/*v*) trypsin-0.91 mM EDTA solution (Gibco) and cultured at a density of 6 × 10^5^ cells/sample as shown below. Culture media of half of the samples were changed for chondrogenic differentiation media (Lonza) supplemented with 5% FBS, 0.2% R^3^-insulin-like growth factor-1 (R^3^-IGF-1), 0.5% transforming growth factor beta 1 (TGF*β*1), 0.2% transferrin, 0.2% insulin, 0.1% gentamicin/amphotericin-B (GA), and 70 mM ascorbic acid.

### 2.2. Agarose Hydrogel Preparation, Cytotoxicity Assay, and Microtissue Formation

**Figure 1:**
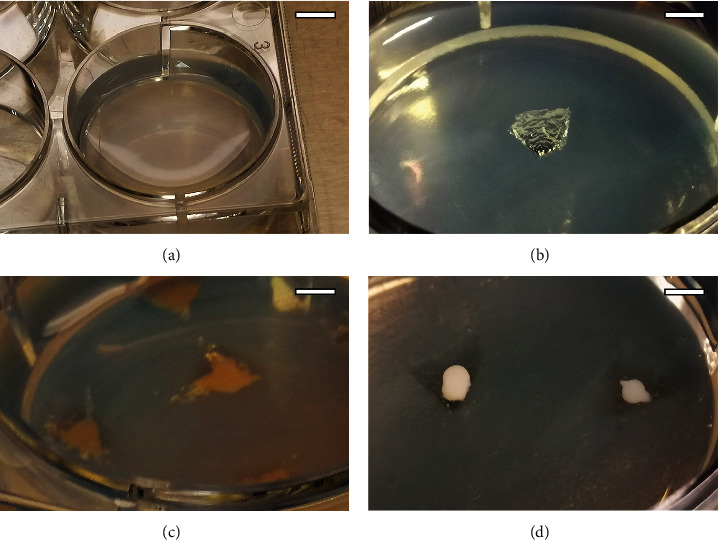
Process of the cell microtissues elaboration in 3% agarose wells. (a) 3% agarose hydrogel after 48 h at 4°C; (b) elaboration of wells in agarose hydrogel; (c) cellular seeding in agarose wells; and (d) completely formed microtissues after 72 h of cell culture. Scale bars—A: 12 mm; B-D: 3 mm.

Hydrogels of 3% type IX-A agarose were prepared as previously described [67], with minor modifications, and allowed to solidify for 48 h (Figure 1(a)). Cytotoxicity of the agarose hydrogel was tested using the MTS assay (CellTiter 96 Aqueous One Solution Cell Proliferation Assay, Promega, Spain). Cell culture medium was conditioned as follows: proliferation medium without phenol red was added to 3% agarose hydrogel at a 1 : 2 *v*/*v* ratio and incubated for 1, 3, and 7 days at 37°C and 5% CO_2_. hDPSCs were seeded in 96-well plates at a density of 10^4^ cells/well. After 24 h of cell culture, the medium was removed and conditioned media were added. Latex-conditioned medium was used as a positive cytotoxic control, and nonconditioned medium was used as a negative control. After 24 h of cell culture, the MTS assay was carried out following the manufacturer's indications.

Wells of 4 mm-diameter and 5 mm-deep were created in 3% agarose hydrogel blocks using a laboratory spoon (Figure 1(b)). Then, 6 × 10^5^ cells were resuspended in 10 *μ*L proliferation culture medium and added to each well created in the agarose (Figure 1(c)); the agarose block was placed in the cell incubator, and the microtissues were allowed to form for 72 h (Figure 1(d)). This was considered the initial, control time (*t* = 0). Samples were cultured in proliferation or chondrogenic differentiation media for 3, 14, 28, and 42 days. The corresponding cell culture medium was changed every 2-3 days.

### 2.3. Histological Studies

Cell morphology and chondral components content were evaluated following standard histological procedures. Briefly, samples were rinsed twice with PBS and fixed with 4% buffered formaldehyde at 4°C for 20 min. Samples were rinsed twice again with PBS and embedded in paraffin (following standard protocols), and 3 *μ*m thick sections were obtained, which were stained with haematoxylin and eosin (H-E) solutions. Stained sections were analysed under an optical microscope (DM 4000B; Leica Biosystems, Germany) and photographed using the Leica DFC 420 camera. Cell density was analysed on stained samples by cellular counting of 10 randomized fields throughout the section, using Image-Pro Plus 6.0 software.

Aggrecan and type I and II collagen content were evaluated by immunofluorescence as previously described [25]. Microtissue sections were deparaffined and rehydrated through graded ethanol and rinsed with PBS, and immunofluorescence was carried out. Specific mouse IgG anti-human antibodies (sc-166951, aggrecan, Santa Cruz Biotechnology, dilution 1 : 200; C2456, type I collagen, Sigma, dilution 1 : 100; and CP18, type II collagen, Millipore, dilution 1 : 500) and secondary anti-mouse-IgG FITC-conjugated antibody (F2883, Sigma, dilution 1 : 200) were used. Some samples were incubated only with the secondary anti-mouse-IgG antibody as control. For nuclei and actin filament staining, samples were incubated with 300 nM DAPI (Sigma) and rhodamine-phalloidin (1 : 200 in PBS) (Invitrogen, USA), respectively, for 2 h at RT. Samples were analysed under a fluorescence microscope (DM 4000B; Leica Biosystems) and photographed using the Leica DFC 340FX camera.

### 2.4. Gene Expression Analysis

Finally, the expression of chondrogenic-related genes in chondrogenic-induced hDPSCs microtissues was evaluated by real-time RT-PCR, as follows. On the one hand, SOX9, ACAN, and COL2A1 are characteristic genes associated with chondrogenesis; on the other hand, COL1A1 is related to fibrous tissues. After microtissue formation, culture medium was replaced by chondrogenic differentiation medium, and samples were cultured for different times: 1, 6,and 24 h and 3, 7, and 14 days. Culture medium was changed every 2-3 days. Then, RNA was isolated using Trizol LS reagent (Ambion, Life Technologies, USA), and RNA was converted to cDNA by reverse transcription PCR using High-Capability cDNA Reverse Transcription Kit (Applied Biosystems, USA). Finally, real-time PCR was performed using primers for the previously mentioned genes and GAPDH as housekeeping gene (Taqman, Applied Biosystems) as shown in Table 1. Relative gene expressions were normalized to GAPDH expression, and the fold change was presented using the ∆∆C_T_ method by comparing to control samples at initial time (without chondrogenic differentiation induction).

### 2.5. Data Presentation and Statistical Analysis

The results shown correspond to two independent experiments, and in each of them, experimental samples were replicated 3 times. The histological study was performed in a double-blinded manner, and the figures presented are representative images.

For statistical analysis, Tukey's test one-way ANOVA was performed to find differences between samples at different timepoints, with *p* value significance threshold of ≤0.05.

## 3. Results

### 3.1. Agarose Hydrogel Cytotoxicity

**Figure 2:**
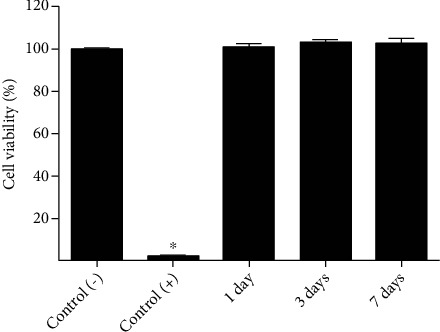
Cell viability assay results. hDPSCs were cultured with media conditioned for 1, 3, and 7 days with 3% agarose hydrogel. Nonconditioned medium and latex-conditioned medium were used as negative and positive cytotoxicity controls, respectively. MTS assay was carried out as described in Material and Methods, and bars show the mean ± SD. ^∗^*p* < 0.001 compared to negative control.

Cell viability of hDPSCs cultured with media conditioned for 1, 3, or 7 days with 3% agarose hydrogels was maximum with no statistically significant differences from that of the negative control, whereas cell viability of hDPSCs cultured with latex-conditioned media (cytotoxicity-positive control) was significantly low, as expected (Figure 2).

### 3.2. H-E Staining

**Figure 3:**
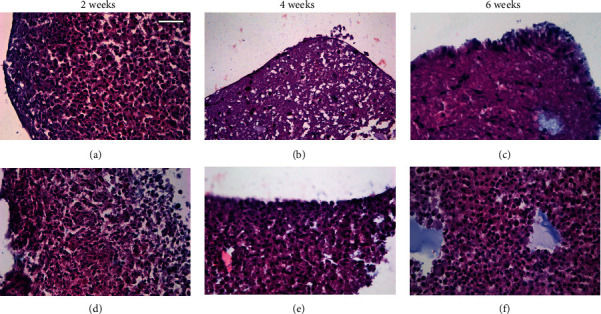
H-E staining images of hDPSC microtissue cultures after (a, d) 2 weeks, (b, e) 4 weeks, and (c, f) 6 weeks. Culture media used were (a–c) proliferation and (d–f) chondrogenic differentiation. Scale bar = 50 *μ*m.

After 2-week culture, no morphological differences were observed between microtissues cultured with either proliferation or chondrogenic differentiation media (Figures 3(a) and 3(d)), showing cells tightly packed and homogeneously distributed throughout the cellular organoid without spaces between cells. At this time, cells showed a polygonal morphology with an eosinophilic cytoplasm and a round or elliptic basophilic nucleus with dense chromatin. Differences were observed at 4 and 6 weeks of cell culture, when samples cultured with proliferating medium showed the presence of a single layer of cells at the organoid periphery, whereas large areas of ECM and a lower density of cells were observed in the center of the organoid (Figures 3(b) and 3(c)). On the contrary, the samples cultured with chondrogenic differentiation medium for 4 and 6 weeks did not show differences from those cultured for 2 weeks with this same medium, and the cells were tightly packed and homogeneously distributed throughout the microtissue, with scarce ECM between them (Figures 3(e) and 3(f)).

### 3.3. Cell Density Quantification

Table 2 shows the cellular density of hDPSC microtissues. At the beginning of the cell culture (*t* = 0), samples showed a density of 9,400 cells/mm^2^. After 3-day culture with proliferation and chondrogenic differentiation media, 12,800 and 12,200 cells/mm^2^ were counted, respectively, with no significant differences between both conditions. In 2-week microtissues cultured with proliferation medium, a slight increase to 13,000 cells/mm^2^ was observed, while in the samples with differentiation medium 10,500 cells/mm^2^ were counted. No statistically significant differences were observed between both conditions. After 4-week culture, proliferation samples decreased to 2,500 cells/mm^2^. On the contrary, chondrogenic differentiation microtissues reached values of 11,600 cells/mm^2^, a cell density significantly different when compared to that of the proliferation samples. Finally, samples maintained for 6 weeks in proliferation culture medium showed cell density values of 4,600 cells/mm^2^, while differentiation samples reached a cell density of 12,100 cells/mm^2^. Significant differences were observed between both conditions. Microtissues cultured in chondrogenic differentiation media did not show significant differences in the number of cells throughout the time of culture.

### 3.4. Immunodetection

Initial time (*t* = 0) and 3-day samples showed a compact formation of the microtissues as previously described. In these cases, samples did not show aggrecan or type II and I collagens synthesis with either proliferation or chondrogenic differentiation culture media (data not shown).

**Figure 4:**
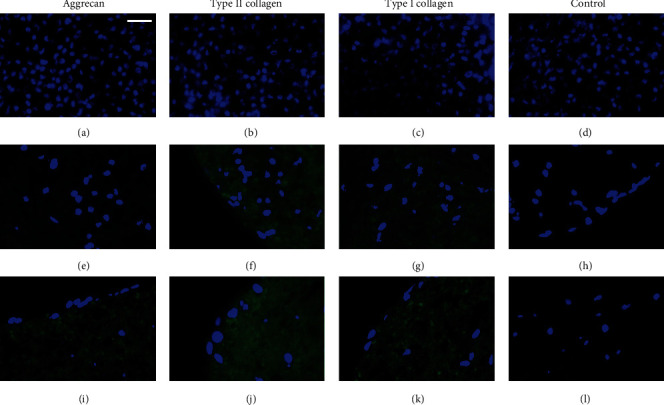
Immunofluorescence microphotographs of hDPSCs microtissue-cultures with proliferation medium for (a–d) 2 weeks, (e–h) 4 weeks, and (i–l) 6 weeks. Control samples were incubated only with the secondary antibody. Cellular nuclei are observed in blue, and the presence of the different specific macromolecules was analysed in green. To facilitate the visualization of the fluorescence signal, green channel parameters of some images were modified by PhotoShop: (e, g) saturation + 100% and luminosity + 50% and (f) saturation + 100% and luminosity + 25%. Scale bar = 25 *μ*m.

Cells cultured with proliferation medium did not show the presence of aggrecan until the 4^th^ week of culture, when small, scattered regions were labelled (Figures 4(a) and 4(e)). This labelling increased at the 6^th^ week of culture, but interestingly, it showed different intensity at the different zones of the microtissue, since it was mainly located at the central zone of the organoid (Figure 4(i)). Synthesis of type II collagen was first observed at the 4^th^ week in the external zone of microtissue, right under the layer of peripheral cells, and it increased in the 6-week culture, when it was homogeneously distributed throughout the organoid (Figures 4(b), 4(f) and 4(j)). Finally, as regards to type I collagen, small areas adjacent to the cell nuclei were labelled at 4-week of culture, while at 6 weeks it was distributed throughout the microtissue (Figures 4(c), 4(g) and 4(k)).

**Figure 5:**
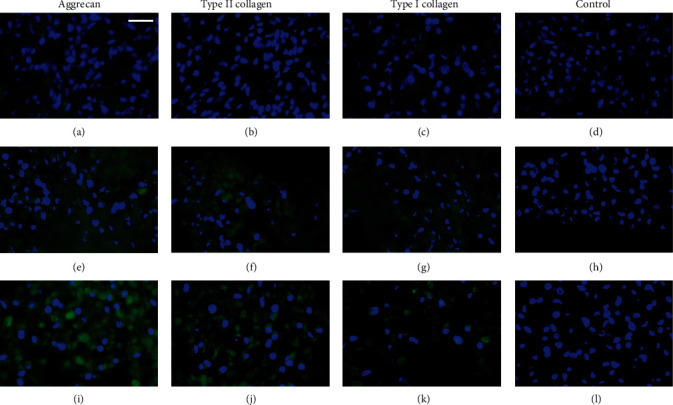
Immunofluorescence microphotographs of hDPSC microtissue cultures with chondrogenic differentiation medium for (a–d) 2 weeks, (e–h) 4 weeks, and (i–l) 6 weeks. Control samples were incubated only with the secondary antibody. Cellular nuclei are observed in blue, and the presence of the different specific macromolecules was analysed in green. To facilitate the visualization of the fluorescence signal, green channel parameters of some images were modified by PhotoShop: (a, c) saturation + 100% and luminosity + 50% and (e–g) saturation + 100% and luminosity + 25%. Scale bar = 25 *μ*m.

In the case of chondrogenic differentiation cell cultures, in all microtissues, there was a homogeneous distribution of the cells throughout the organoids, as mentioned above. As expected, aggrecan synthesis was observed earlier than in those cells cultured in proliferation medium, since it was detected from the 2^nd^ week, increased over time, and became abundant at 6 weeks (Figures 5(a), 5(e) and 5(j)). The presence of type II collagen was observed at 4-week cell culture, increasing throughout the 6 weeks of the microtissue culture (Figures 5(b), 5(f) and 5(j)). Finally, type I collagen labelling was also slightly observed at 2-week culture, especially around cell nuclei (suggesting intracellular location), and increased mildly its synthesis at 4 and 6 weeks (Figures 5(c), 5(g) and 5(k)).

### 3.5. Gene Expression Analysis

**Figure 6:**
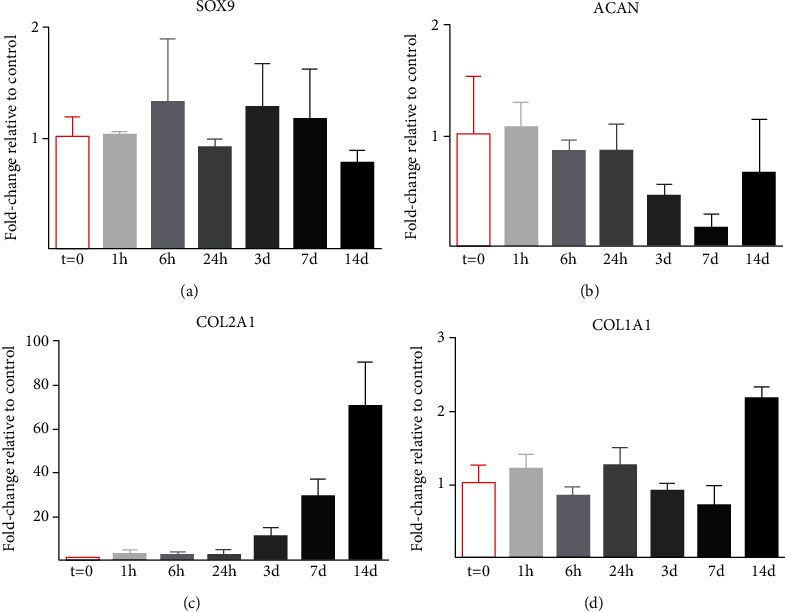
Relative expression of (a) SOX9, (b) ACAN, (c) COL2A1, and (d) COL1A1 genes after 1, 6, and 24 h and 3, 7, and 14 days of chondrogenic differentiation medium culture of hDPSC microtissues. Gene expression was normalized to GAPDH expression and compared to control samples (*t* = 0). Mean ± SD are shown. Statistical significance at ^∗^*p* ≤ 0.05 with respect to control.

Histological results showed a lower cell density and a peripheral cell distribution in microtissues cultured in proliferation medium. Furthermore, as expected, chondral components were increased in microtissues cultured with differentiation rather than with proliferation media. Thus, the former microtissues were chosen for further analysis of the gene expression. Since gene expression occurs earlier than protein synthesis, short periods of culture were included in this study. The analysis of the expression of gene markers was studied in microtissues cultured for short periods (1, 6, and 24 h) and longer periods (3, 7, and 14 days) with chondrogenic differentiation medium. Significant differences on COL2A1 and COL1A1 gene markers were observed. COL2A1 gene expression showed a 10-fold increase with respect to control on the third day of culture (Figure 6(c)). This increase was close to a 30-fold change after 7 days of culture and over a 70-fold change after 14 days. Besides, COL1A1 gene expression showed a 2-fold increase after 14 days of chondrogenic differentiation culture (Figure 6(d)). No significant differences were observed for SOX9 and ACAN gene expressions throughout the time of culture studied (Figures 6(a) and 6(b)).

## 4. Discussion

Since cartilage has a low regenerative capability [7, 8], a high number of therapies have emerged to solve chondral pathologies. These therapies have achieved acceptable results, but mainly result in the formation of fibrocartilage [10]. For this reason, tissue engineering approaches do not focus on repairing but on regenerating these injuries [1]. Different types of cells have been studied over the years, and researchers have demonstrated that MSCs [27, 28], such as DPSCs and ADSCs, among others, can differentiate to the chondrogenic lineage [36]. Moreover, 3D environments such as PLA scaffolds or hydrogels have been used to culture cells in them, improving cell differentiation and showing good results considering the synthesis of proteins characteristic of the chondral extracellular matrix [50, 51]. Finally, it is known that growth factors can promote an earlier chondrogenic differentiation of cells in culture [47].

In this work, hDPSC microtissues were generated and kept in 3% agarose wells. The results obtained using cell culture media conditioned with agarose hydrogel demonstrated that this hydrogel was not cytotoxic, as described by others [68, 69]. The microtissues formed were cultured for several weeks, with either proliferation or chondrogenic differentiation media. After that, the content of aggrecan and type II and I collagens was analysed, and the different culture conditions were compared to study the chondrogenic differentiation potential of this type of mesenchymal stem cell.

We observed that cells forming microtissues synthesized and secreted aggrecan and type II collagen and, to a lesser extent type I collagen, either in the presence of proliferation medium or chondrogenic differentiation medium, in the latter at shorter times of cell culture. These results agree with those of Mahboudi et al., where significant deposition of aggrecan and increased COL2A1 gene expression were detected in pelleted cultures of induced pluripotent stem cells (iPSCs) [70]. Therefore, cell-cell proximity, occurring in these microtissues, induced the production of macromolecules of the extracellular chondral matrix, obtaining better results than in studies where cells were cultured in a hydrogel-scaffold matrix [52, 65, 66, 71].

HE staining and cell number quantification showed differences in both cell density and distribution when comparing microtissues cultured with proliferation or differentiation media for several weeks. In the former, microtissue cell density was lower and cell was distributed mainly on the surface, while in the latter cell distribution was homogenous across the microtissue volume. It is tentative to consider that the different ECM composition, as deduced from the HE staining and immunofluorescence results, interferes or makes the growth of the cells in the central zone of microtissues cultured with proliferation medium more difficult. Considering these results, we decided to study the expression of selected genes in microtissues cultured in differentiation medium. We observed that the expression of COL2A1 exhibited an early induction when hDPSC microtissues were exposed to chondrogenic differentiation medium, which agrees with the results of Zhang et al., which demonstrated the chondrogenic potential of MSC microtissues exposed to a chondrogenic inducing medium [54].

It has been demonstrated that a 3D cell niche is important to improve chondrogenic differentiation of MSCs, as DPSCs [72], and many research studies have focused on developing and optimising 3D architectures for that purpose. Several polymers, such as PLA (polylactic acid), PCL (polycaprolactone), PEGMA (poly(ethylene glycol)methacrylate), and gelatine, among others, have been used to create suitable hydrogels or nanofiber-based scaffolds with adequate physical properties to promote chondrogenic differentiation [72–75]. However, also numerous studies are currently focused on the development of organoids, creating organomimetic tissues [67, 76–78], which mimic and improve the functions of the native tissues where they will be implanted. Our results agree with those of Torras et al., who reported that pluripotent cells, as DPSCs, have a high capability for intrinsic organization when cultured in an organoid or microtissue model, which is related to different cell functions, such as extracellular matrix synthesis, among others [76].

Thus, we have observed that the chondrogenic differentiation medium used promoted the early synthesis of these components, mainly aggrecan and type II collagen, while maintaining stable architecture of the microtissues. These results agree with the studies by Meyer-Wagner et al., where cell organoids exposed to electromagnetic fields (EMF) and cultured with chondrogenic differentiation medium showed a higher expression of gene characteristic of the chondral lineage than those cells exposed to EMF and cultured with cellular expansion medium [79].

Therefore, not only the cell culture medium but also the 3D environment is essential in chondrogenic differentiation to obtain good results considering the synthesis of components of the chondral matrix such as aggrecan and type II collagen. In this work, hDPSC microtissues cultured with chondrogenic differentiation medium promoted the onset of chondral components at shorter times than the same cells cultured with proliferation medium, which is an important point to improve the regeneration of articular chondral injuries.

## 5. Conclusions

The choice of the cell type, the culture environment, and the media are critical to obtain good results in biomedical studies. Firstly, we have shown that hDPSCs have a high chondrogenic potential. Secondly, a 3D environment, such as microtissues or organoids, favours the chondrogenic differentiation of cells. Finally, culture media with suitable growth factors, such as those present in the chondrogenic differentiation medium, can accelerate the synthesis of different macromolecules characteristics of the extracellular matrix of the hyaline articular cartilage, such as aggrecan and type II collagen.

We are establishing the experimental conditions for grafting hDPSCs microtissues in an animal model, to evaluate in the near future their in vivo chondrogenic differentiation potential. Furthermore, we are currently studying the effect of magnetic irradiation and mechanical stimuli on the chondrogenic differentiation of hDPSC microtissues formed as described herein.

## Figures and Tables

**Table 1:** Gene marker primers.

Gene	ID reference	Function
SOX9	Hs00165814_m1	Chondrogenic transcription factor
ACAN	Hs00153936_m1	Hyaline cartilage-related ECM component
COL2A1	Hs00264051_m1	Hyaline cartilage-related ECM component
COL1A1	Hs00164004_m1	Fibrous cartilage-related ECM component
GAPDH	Hs99999905_m1	Housekeeping gene

**Table 2:** Cell density (cells/mm^2^) of hDPSC microtissues cultured with proliferation or chondrogenic differentiation media for 3 days and 2, 4, and 6 weeks. Samples at initial time (*t* = 0; cell density: 9,400 ± 600) were taken as control. Values shown (mean ± SD) are ×10^3^. Statistical significance at *p* ≤ 0.05 with respect to control (^∗^) and to samples cultured with proliferation medium for the same period of time (^#^).

	3 days	2 weeks	4 weeks	6 weeks
Proliferation	12.8 ± 0.4^∗^	13.0 ± 1.3^∗^	2.5 ± 0.4^∗^	4.6 ± 0.8^∗^
Chondrogenic differentiation	12.2 ± 0.7∗	10.5 ± 0.1^∗^	11.6 ± 0.6^∗,#^	12.1 ± 1.3^∗,#^

## Data Availability

The data used to support the findings of this study are included within the article.
